# Numerical Analysis of the Porous Structure of Activated Carbons Derived from Synthetic Polymers

**DOI:** 10.3390/ma17133122

**Published:** 2024-06-26

**Authors:** Mirosław Kwiatkowski, Isabel Pestana da Paixão Cansado, Paulo Mira Mourão

**Affiliations:** 1 Faculty of Energy and Fuels, AGH University of Krakow, al. Adama Mickiewicza 30, 30-059 Krakow, Poland; 2 MED—Mediterranean Institute for Agriculture, Environment and Development, CHANGE—Global Change and Sustainability Institute, Department of Chemistry and Biochemistry, School of Science and Technology, University of Évora, Rua Romão Ramalho 59, 7005-671 Évora, Portugal; ippc@uevora.pt (I.P.d.P.C.); pamm@uevora.pt (P.M.M.)

**Keywords:** activated carbon, activation, polyethylene terephthalate, polyacrylonitrile

## Abstract

This paper presents original results from the unique analysis of the porous structure of activated carbons (ACs) produced through the chemical activation of polyethylene terephthalate (PET) and polyacrylonitrile (PAN), as well as from a physical mixture of both polymers. An advanced method of adsorbent surface analysis—more specifically, the new method of numerical clustering-based adsorption analysis regarding the surface heterogeneity, pore geometry and adsorption energy distribution parameters—allowed us to obtain information about the porous structure of the ACs from the synthetic polymers mentioned above. As the results showed, ACs obtained with PAN were characterised by a first adsorbed layer with the highest volume. When the surface heterogeneity, highly desirable in most advanced adsorption processes, is taken into account, the materials with the best surface properties in both potassium carbonate (K_2_CO_3_) and potassium hydroxide (KOH) activation processes were the ACs obtained with a mass proportion of PET to PAN of 1:3, which were characterised by a low degree of surface heterogeneity and a first adsorbed layer presenting a relatively large volume.

## 1. Introduction

In recent years, industry and the global economy have concluded that economics and ecology are not contradictory, and environmental protection is also necessary from an economic point of view [1]. Consistent protection of inland waters, soil and air is an indispensable premise for sustainable economic development in the long run [2]. Therefore, many international initiatives have led to the strengthening of efforts and research to protect the natural environment and reduce emissions of pollutants into the environment [3]. Such ecological and energy problems, including the need to reduce the emission of harmful substances to the environment, have increased interest in new solutions for environmental protection [4,5]. These new solutions are based, among others, on the use of adsorption processes on microporous activated carbons [6,7,8,9,10], which are amorphous carbonaceous materials characterised by a well-developed porous structure and thus a large specific surface area.

The activated carbons can be produced through direct activation of the raw materials or by using a two-step process involving initial carbonisation followed by physical activation or even by chemical activation [11]. The process of carbonisation is based on the thermal treatment of raw materials made in an inert gas environment and subsequent activation [12,13]. Normally, physical activation takes place in the presence of steam or carbon dioxide, usually within the temperature range from 800 to 1000 °C [14]. The use of steam as an activate agent results in the development of wider micropores and mesopores with a wider pore size distribution, while the use of carbon dioxide (CO_2_) promotes mainly the development of ultra micropores, which presents a fine pore size distribution [14,15].

The temperature at which the activation takes place is a determinant in the development of the porous structure, so it should be carefully selected and controlled [16]. Activation is an endothermic process. Since lower activation temperatures could adversely affect the kinetics of the reaction, activation should be taking place at relatively high temperatures. At the appropriate temperature, the activation process reactions take place on the internal surface of the carbonaceous material, and during these reactions, the carbon is removed from the inside of the pores, which causes their widening. However, too high temperatures can burn the external surface of the carbonaceous particles.

Even small amounts of activating agents can speed up the process during physical activation, promoting a high burn-off of the material. Industrially, KOH and K_2_CO_3_ are often added as catalysts in the physical activation process [17]. Physical activation presents some advantages, such as low cost and the ability to retain the texture and shape of the precursor.

Chemical activation, on the other hand, involves heating at high temperatures the raw material or char impregnated with a suitable chemical agent such as NaOH, KOH, ZnCl_2_, H_3_PO_4_ or FeCl_3_, among others [18]. In the case of activation using NaOH or KOH, a preliminary carbonisation step is recommended; without it, the strong bases may destroy the organic fraction of the raw material, making it impossible to be activated [19]. The mass relation between the activating agent and virgin raw material and the activation temperature are among the most important parameters influencing on the creation of ACs and their porosity during chemical activation [18,19].

Chemical activation has several benefits, i.e., it makes it possible to carry out the process at inferior temperatures, has higher process yields and lower process costs due to lower heat treatment temperatures and shorter process times, and allows, above all, for obtaining materials with very large surface areas and very well-developed porosity and narrow pore size distributions [18,19]. However, the main disadvantage of chemical activation is the need for chemicals that can be corrosive or toxic, as well as the additional step of washing the final product to remove activator residues, which increases the cost of the overall activated carbon manufacturing process and poses an environmental risk.

With the selection of proper raw materials, suitable preparation procedures and correct control of the production settings, the textural properties of ACs can be dimensioned to specific requests. Theoretically, any substance with a high content of carbon and low ash fraction can be used as a raw material for ACs production. The need for porous materials with high mechanical strength and large specific surface area for applications in power engineering and environmental protection has led to research development in recent years, particularly on ACs production from waste synthetic polymers [20,21,22,23,24,25].

Activated carbons produced from synthetic polymers are particularly suitable when low-ash adsorbents are required. In particular, polyethylene terephthalate (PET) is an excellent precursor because of its high carbon content (62.5%) [25], as well as its ability to effectively manage the common waste materials made from this plastic. However, the low pyrolysis yield of PET during AC production is a disadvantage in its use. To improve the PET pyrolysis yield, its co-activation with other polymers such as polyacrylonitrile (PAN) is recommended [25]. Polyacrylonitrile is a commonly used raw material for the production of activated carbon fibres (ACF) and carbonaceous materials since it has a comparatively high efficiency in the carbonisation process.

Therefore, to obtain improved activated carbons, the raw material was co-activated with bituminous coal or other polymers, including both natural and synthetic ones [25]. One polymer that can be added successfully to a blend with PET is polyacrylonitrile (PAN), which is widely used in the manufacture of carbon fibres and other carbonaceous materials due to its relatively high carbon content [25].

Using the aforementioned polymers to produce activated carbons also reduces the waste stream that directly pollutes the natural environment, transforming them into useful adsorption materials commonly used in environmental protection processes. This solution is also competitive with other methods of dealing with plastic waste based on unproductive landfilling and the controversial method of thermal disposal.

To date, however, a small number of publications [25] have presented the results of work devoted to obtaining activated carbons from a mixture of PET and PAN, so there is a need for this type of research work.

## 2. Materials and Methods

Cansado et al., 2019 [26], presented some results concerning the preparation of ACs from polyethylene terephthalate (PET) and polyacrylonitrile (PAN) as well as from a physical mixture of both polymers. The preparation of activated carbons was carried out via the activation with K_2_CO_3_ or KOH by heating the mixture at a rate of 10 K min^−1^ until reaching 1073 K [26]. The ACs were labelled as follows: in the first part, the letters stand for the abbreviation of the name of the raw material, i.e., PET and PAN, and then the mass ratio of activated carbon precursors is given. For example, the label PET:PAN-1:3 of an activated carbon sample means that it is a sample of material obtained from PET and PAN in a mass ratio equal to 1:3.

Nitrogen adsorption isotherms for ACs were obtained at 77 K on a Quadrasorb, Quantachrome Instruments. Before obtaining adsorption isotherms, ACs were degassed for 5 h at 573 K, using a rate of 2 K min^−1^ [26]. Based on the determined adsorption isotherms, *A_BET_* was assessed using the Brunauer–Emmett–Teller (BET) method [27], total micropore volume *V_s_* was calculated using the α_s_ method and micropore volume *V*_0_ and average micropore width *L*_0_ were found using the Dubinin–Radushkevich (DR) method [28].

To correctly select the method and experimental conditions used to produce activated carbons, the adsorption properties of the activated carbons obtained and the textural properties must be determined with relative precision. As the physical adsorption of gases and vapours is a popular method for analysing the porous structures of adsorbents, various equations have been developed to describe the rapport between the amount of gas or vapour adsorbed and the relative pressure of the adsorptive. Firstly, these equations were simple empirical formulas, but with advances in research into the mechanisms of adsorption processes, thermodynamic equations were developed based on physical models. Among other things, the commonly used Brunauer–Emmett–Teller (BET) equation based on a much-simplified model of the multilayer adsorption process shows many limitations and imperfections when analysing real adsorption processes on heterogeneous materials, especially when analysing N_2_ adsorption isotherms [29].

The Dubinin–Radushkevich (DR) equation is also used to describe gas adsorption, mainly in micropore structures. Firstly, the DR equation was defined empirically, but based on the theory of the micropore filling process proposed by Dubinin and Radushkevich [28]. A common problem with this equation is that the adsorption isotherms obtained in many microporous carbon materials do not fit into the DR equation. In addition, the effect of temperature on the microporosity volume values obtained may become apparent due to the presence of submicropores.

The models of the adsorption process and the BET and DR equations derived from them have significant simplifications and limitations, one of the most important being the omission of surface heterogeneities and interactions between adsorbed molecules [29]. As emphasised in the literature, all real adsorbents exhibit energy heterogeneities, thus showing a distribution of adsorption potentials, significantly affecting the adsorption properties; therefore, determining the degree of surface heterogeneity is one of the key issues [30,31]. However, the description of heterogeneous surfaces additionally poses significant problems in terms of quantifying the phenomena that take place on the adsorbent surface. Consequently, various attempts have been made to develop reliable methods to define the process taking place during adsorption. One such method is a unique proprietary method for the analysis of cluster-based multilayer adsorption processes on heterogeneous surfaces (LBET), together with an advanced fast numerical method for multi-variant identification of adsorption systems [32,33,34].

The LBET method is based on a group of 30 or, in an extended version, 48 models and a unique universal adsorption theory developed by the team, which is a significant extension of the BET model. In addition, adsorption in ultramicropores is described by the equation of Langmuir, thence the abbreviation LBET is used for the newly proposed group of mathematical models of the adsorption process [32,33,34]. In contrast to the BET model, the LBET model was developed to consider the heterogeneity of the surface and the possibility of separating groups of adsorbate molecules, as well as geometrical and energetic constraints on the formation of groups of molecules from the adsorbate [32,33,34]. According to the theory on which the LBET method is based, adsorbate molecules adsorb in micropores, forming clusters whose size is controlled by the form of the pores or the competitive extension of neighbouring clusters. Adsorbate molecules that have been adsorbed as a result of adhesion interactions with the adsorbent surface are considered to be the first adsorbed layer. The attachment of further molecules to those originally adsorbed in LBET theory is treated as the establishment of second, third and subsequent adsorption layers [32,33,34]. Two classes of LBET models are implemented in the LBET method, i.e., a model where the number of adsorption layers is controlled by the competitive expansion of adjacent groups of adsorbate molecules, and a second model where adsorption is described as a system in which the restrictions on the expansion of groups of adsorbate molecules are due to the geometrical constraints of the porosity. The LBET models can be described based on five parameters: the volume of the first adsorbed layer *V_hA_* (cm^3^/g), the dimensionless energy parameter for the first adsorbed layer *Q_A_*/*RT*, the dimensionless energy parameter for the higher adsorbed layers *B_C_*, the geometrical parameter of the porous structure determining the height of the adsorbate molecule clusters *α* and the geometrical parameter of the porous structure determining the width of the adsorbate molecule clusters *β*, which can be determined by fitting the LBET model equation to an experimental adsorption isotherm, with a selected variant of the adsorption energy distribution function on the adsorbent surface [32,33,34].

The numerical determinants of system identification tasks are strongly impacted by the energy heterogeneity of microporous adsorption systems. To fit LBET class models to adsorption isotherms, a special fast multi-variant numerical method has been developed. This method is also utilised to ascertain the shape of the adsorption energy distribution on the first layer and the value of the surface heterogeneity index, *h* [32,33,34]. Since, as mentioned, the popular BET and DR methods do not provide the required information concerning the ACs porous structure, to appropriately select the method and conditions for their production process, the concept of carrying out new unique analyses of the ACs’ textural properties and the adsorption process occurring on their surface using an innovative analysis method taking into account surface heterogeneity and pore geometry, i.e., the LBET method described above, was developed.

## 3. Results and Discussion

**Figure 1:**
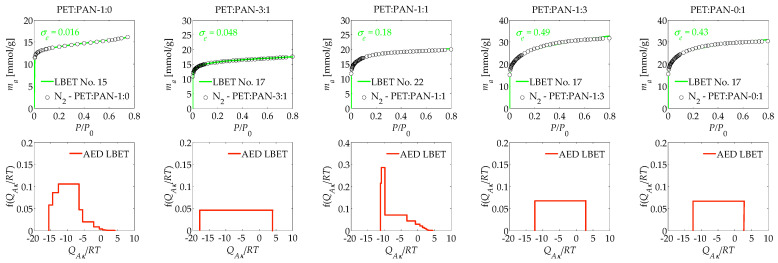
The results of the analysis of a porous structure of ACs obtained via K_2_CO_3_ activation, using the analysis of the N_2_ isotherms, based on the LBET method. The drawings in the first row show the nitrogen adsorption isotherms determined at 77 K and best-fitting LBET models, while the drawings in the second row present the distribution of the adsorption energy on the first layer on the corresponding samples.

**Figure 2:**
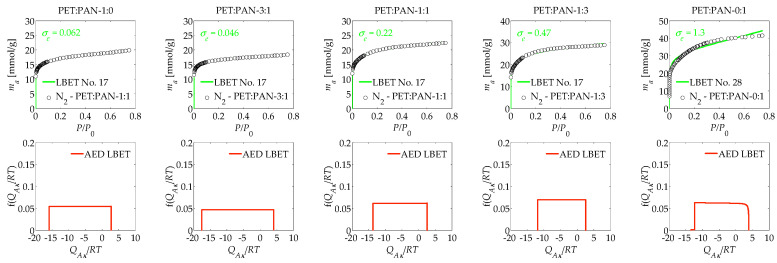
The results of the analysis of a porous structure of activated carbons obtained via KOH activation, based on nitrogen adsorption isotherms, using the LBET method.

The results of the analysis calculations are summarised in Table 1 and Table 2, as well as in Figure 1 and Figure 2. The first activated carbon sample analysed was obtained by activation of K_2_CO_3_ from just polyethylene terephthalate (PET), which was designated as PET:PAN-1:0. This material was characterised by limitations on the growth of groups of adsorbate molecules related to the expansion of adjacent clusters as indicated by the type of best-fitting LBET class model, as well as very high surface heterogeneity, as indicated by the value of the parameter *h* (*h* = 9). In turn, the value of the volume of the first adsorbed layer (*V_hA_*) indicates a relatively small volume, the smallest among the analysed ACs produced through chemical activation with K_2_CO_3_.

The values of parameters *α* and *β* indicate that significantly branched clusters of adsorbate molecules are formed at an average height in the pores of the analysed sample, and the values of dimensionless energy parameters, i.e., *Q_A_*/*RT* and *B_C_* (*Q_A_*/*RT* = −15.57 and *B_C_* = 7.73), indicate preferential environments for multilayer adsorption. The theoretical isotherm fits very well with the empirical data, as can be seen by the parameter *σ_e_* (*σ_e_* = 0.016) and the high identifiability of the adsorption system, confirmed by the *w_id_* parameter (*w_id_* = 0.64). The shape of the adsorption energy distribution (AED) determined for the PET:PAN-1:0 sample obtained from polyethylene terephthalate PET by activation with K_2_CO_3_ indicates the predominant influence on the total surface area of a wide spectrum of sites with a medium adsorption energy value.

The next sample analysed was PET:PAN-3:1 activated carbon obtained with a PET to PAN ratio of 3 to 1. This material was also characterised by a homogeneous surface area (*h* = 1) and an average volume of the first adsorbed layer (*V_hA_* = 0.669 cm^3^/g). In the porosity of PET:PAN-3:1, monolayer branching clusters of adsorbate molecules were formed, which can be confirmed by the geometrical parameters (*α* = 0.06 and *β* = 3.00). This conclusion is also supported by the energy parameter values, which indicate the existence of conditions for the monolayer adsorption processes (*Q_A_*/*RT* = −17.87 and *B_C_* = 4.41). The shape of the AED diagram obtained for the sample PET:PAN-1:0 indicates the presence of extensive energy sites for primary adsorption.

Another material analysed was PET:PAN-1:1 activated carbon, obtained by activation of K_2_CO_3_ with polyethylene terephthalate (PEN) and polyacrylonitrile (PAN) at a mass relation of 1:1. The aforementioned activated carbon was characterised by limitations on the formation of groups of adsorbate molecules correlated to geometrical pore constraints, as shown by the number of the best-fitting LBET model. The mentioned material also has a significant degree of heterogeneity on its surface, as indicated in turn by the *h* parameter (*h* = 5). The value of the *V_hA_* parameter obtained for PET:PAN-1:1 activated carbon indicates the average volume of the first layer adsorbed on the surface of this material (*V_hA_* = 0.618 cm^3^). The values of *α* and *β* obtained (*α* = 0.72 and *β* = 1.00) and the energy parameters indicate preferential conditions for the formation of non-branching clusters of adsorbate molecules in the pores of PET:PAN-1:1 sample and that the multilayer adsorption process take place (*Q_A_*/*RT* = −11.18 and *B_C_* = 7.74). The good match between the theoretical isotherm and the empirical isotherm is remarkable, as is the good traceability of the system, and thus the high reliability of the results obtained. On the other hand, the analysis of the shape of the AED diagram determined for the PET:PAN-1:1 sample indicates the predominance of high-energy sites in the structure of the analysed material and a significant proportion of medium-adsorption energy sites.

The subsequent material analysed was activated carbon obtained at a PET:PAN mass ratio of 1:3, designated as PET:PAN-1:3. For this material, the number of the best-fitting LBET model, i.e., 17, is consistent with the presence of restrictions on the formation of clusters of adsorbate molecules related to the competitive growth of neighbouring clusters. The aforementioned material was identified as having a homogeneous surface structure (*h* = 1) and a very large volume attributed to the first adsorbed layer, the largest between the analysed ACs obtained from a blend of PET and PAN through chemical activation with K_2_CO_3_ (*V_hA_* = 1.125 cm^3^/g). On the porous structure of this material, low and non-branching clusters of adsorbate molecules have formed, and the values of the energetic parameters indicate preferential conditions for the occurrence of monolayer adsorption. Noteworthy is the average identifiability, which may indicate the presence of a structure that deviates from the assumptions of the model construction adopted in LBET theory.

The last activated carbon sample analysed, obtained using K_2_CO_3_ as an activator, was a sample obtained from PAN alone and labelled PET:PAN-0:1. This sample was characterised by cluster growth constraints related to the competition caused by the growth of the adjacent nitrogen molecule clusters and a homogeneous surface area (*h* = 1). For this sample, the volume attributed to the first adsorbed layer was very high (*V_hA_* = 1.125 cm^3^/g), and very low and non-branching groups of molecules of the adsorbate were formed in the porous structures of this material, as indicated by the values *α* and *β* (*α* = 0.13 and *β* = 1.00). On the other hand, on PET:PAN-0:1, the conditions for monolayer adsorption are predominant, which can be confirmed by the energy parameters (*Q_A_*/*RT* = −12.47 and *B_C_* = 1.00), but the average identifiability of this adsorption system is noteworthy, indicating the existence of some nonconformities with the theoretical assumptions of the LBET method. The shape of the AED diagram determined for the sample PET:PAN-0:1 is consistent with the presence of primary adsorption sites, with a wide energy range.

The work also analysed the porous structure of activated carbons analogously obtained from PET and PAN, but this time, potassium hydroxide (KOH) was the activating agent, and the results are presented in Table 2 and shown in Figure 2. The first activated carbon analysed, which was obtained from PET by KOH activation and labelled PET:PAN-1:0, was characterised by limitations on the formation of groups of adsorbate molecules related to geometrical pore constraints, as can be seen by the best-fitting LBET model. This sample was also characterised by some surface homogeneity, as shown by the *h* parameter value (*h* = 1). The value of the *V_hA_* parameter (*V_hA_* = 0.680 cm^3^/g), in turn, indicates the relatively small volume of the first absorbed layer, the smallest among the analysed ACs obtained using KOH as an activating agent.

On PET-PAN-1:0, the geometrical parameters *α* and *β* (*α* = 0.12 and *β* = 1.29) indicate that very low and branching clusters of adsorbate molecules form in its pores, while the energy parameters, i.e., *Q_A_*/*RT* and *B_C_* (*Q_A_*/*RT* = −15.95 and *B_C_* = 1.00), allow for identifying the presence of monolayer adsorption. A very good fit was identified between the theoretical isotherm and empirical data, as confirmed by the value of the parameter (*σ_e_* = 0.062) and the good identifiability of the adsorption system, shown by the *w_id_* index (*w_id_* = 0.24). The shape of the AED diagram for the first layer of adsorbed determined on PET:PAN-1:0 indicates the presence of a wide energy range of primary adsorption sites.

Next, the activated carbon sample labelled PET:PAN-3:1 was analysed, for which significant decreases in the volume of the first adsorbed layer (*V_hA_* = 0.706 cm^3^/g) and in the height of the adsorbate molecule clusters to a height of one molecule (*α* = 0.05) were found, while there was a significant increase in the dimensions of the adsorbate molecule clusters (*β* = 3.00) and on the adsorption energy values on all layers (*Q_A_*/*RT* = −17.60 and *B_C_* = 4.14). Noteworthy is the very good fit between empirical and theoretical isotherms data as well as the very good traceability obtained for the PET:PAN-3:1 sample through KOH activation.

The next activated carbon sample analysed, obtained by KOH activation, was the sample PET:PAN-1:1, which was characterised by a larger volume of the first adsorbed layer *V_hA_* (*V_hA_* = 0.815 cm^3^/g), a larger *α* (*α* = 0.14) and at the same time a lower *Q_A_*/*RT* (*Q_A_*/*RT* = −13.69). 

The succeeding sample analysed was activated carbon designated PET:PAN-1:3, obtained from PET and PAN at a mass ratio of 1 to 3 using KOH as the activator. This sample was characterised by an even larger volume of the first adsorbed layer *V_hA_* (*V_hA_* = 1.100 cm^3^/g), a lower value of the geometrical parameter *α* (*α* = 0.12) and a significantly lower *Q_A_*/*RT* value of (*Q_A_*/*RT* = −11.89) compared to the previously described samples.

The last material analysed in this study was activated carbon PET:PAN-0:1, obtained from polyacrylonitrile (PAN) itself activated with KOH, at 1073 K. The number of the best-fitting model to the nitrogen adsorption isotherm determined on this material indicates the limitations of adsorbate cluster growth related to the geometrical constraints of the pores. To the activated carbon PET:PAN-0:1 was attributed a very high degree of heterogeneity surface (*h* = 9) and the largest volume of the first adsorbed layer among the analysed activated carbons (*V_hA_* = 1.507 cm^3^/g). The values of *α* and *β* obtained (*α* = 1.00 and *β* = 1.00) allow us to state that, very high non-branching clusters of adsorbate molecules form in the pores structure What is notable, however, is the average fit of the theoretical LBET isotherm to the empirical isotherm and thus the poor identifiability of the adsorption system.

## 4. Conclusions

The results of the research presented in this article have provided unique and valuable information on the feasibility of producing activated carbons from synthetic polymers, including waste materials, thus filling a significant gap in global science in this area. This is because, for the first time, the results of the research were presented, and a complete analysis of the activated carbons porous structure was performed. ACs were produced from PET and PAN mixtures through chemical activation with KOH and K_2_CO_3_ and an innovative method of analysing their porous structure, considering the energetic and geometric heterogeneity of ACs surfaces tested.

The results obtained from the calculations and analyses carried out using the new numerical clustering-based adsorption analysis method demonstrated a significant influence of the activating agent and the mass proportion of PET to PAN on the porous structure of the ACs produced. The study showed a significant influence of the raw material on the development of the porous structure and the energetic and geometric heterogeneity of the ACs surface.

Concerning the activated carbons produced through chemical activation with K_2_CO_3_, the sample obtained from pure PET presented the highest degree of surface heterogeneity, while for ACs obtained by chemical activation with KOH, the material with the highest degree of surface heterogeneity was the sample obtained from PAN alone. In contrast, the largest values for the volume of the first adsorbed layer were characterised by activated carbon samples obtained from pure PAN regardless of the chemical activator used. Taking the surface homogeneity desirable in most advanced adsorption processes as a criterion, the materials with the best adsorption properties in both potassium carbonate and potassium hydroxide activation processes turned out to be activated carbons obtained at a mass ratio of PET to PAN of 1:3, which were characterised by a low degree of surface heterogeneity and, at the same time, a large volume of the first adsorbed layer. In conclusion, it should be emphasised that the research carried out has provided clues for technologists, which, in the context of the possibility of managing synthetic polymer waste, which is common worldwide, is of great importance for sustainable development and in line with the principles of the circular economy.

## Figures and Tables

**Table 1:** Calculation results forthe ACs produced through K_2_CO_3_ activation at 1073 K, based on nitrogen adsorption isotherms, using the LBET method.

Activated Carbon	Model LBET No.	*h*	*V_hA_* (cm^3^/g)	*α*	*β*	*Q_A_*/*RT*	*B_C_*	*σ_e_*	*w_id_*
PET:PAN-1:0	15	9	0.448	0.48	2.46	−15.57	7.73	0.016	0.64
PET:PAN-3:1	17	1	0.669	0.06	3.00	−17.87	4.41	0.048	0.60
PET:PAN-1:1	22	5	0.618	0.72	1.00	−11.18	7.74	0.18	0.43
PET:PAN-1:3	17	1	1.125	0.22	1.00	−12.37	1.00	0.49	0.21
PET:PAN-0:1	17	1	1.139	0.13	1.00	−12.47	1.00	0.43	0.27

*h* is the surface heterogeneity parameter; *V_hA_* is the volume of the first layer adsorbed; *α* is the porous structure geometrical parameter, which allows the determination of the height of the adsorbate molecule clusters; *β* is the porous structure geometrical parameter, which allows the determination of the width of the adsorbate molecule clusters; *Q_A_*/*RT* is the dimensionless energy parameter for the first layer adsorbed; *B_C_* is the dimensionless energy parameter for the higher layers adsorbed; *σ_e_* is the dispersion value of the fit error; *w_id_* is the adsorption system identifiability index.

**Table 2:** Calculation results for the ACs produced through KOH activation at 1073 K, based on nitrogen adsorption isotherms, using the LBET method.

Activated Carbon	Model LBET No.	*h*	*V_hA_* (cm^3^/g)	*α*	*β*	*Q_A_*/*RT*	*B_C_*	*σ_e_*	*w_id_*
PET:PAN-1:0	17	1	0.680	0.12	1.29	−15.95	1.00	0.062	0.24
PET:PAN-3:1	17	1	0.706	0.05	3.00	−17.60	4.14	0.046	0.55
PET:PAN-1:1	17	1	0.815	0.14	1.00	−13.69	1.00	0.22	0.18
PET:PAN-1:3	17	1	1.100	0.12	1.00	−11.89	1.00	0.47	0.13
PET: PAN-0:1	28	9	1.507	1.00	1.00	−13.48	4.03	1.3	0.09

## Data Availability

The data presented in this work can be made available on request.
